# Diuretics: a review of the pharmacology and effects on glucose homeostasis

**DOI:** 10.3389/fphar.2025.1513125

**Published:** 2025-03-28

**Authors:** Mauricio Di Fulvio, Yakshkumar Dilipbhai Rathod, Shorooq Khader

**Affiliations:** Department of Pharmacology and Toxicology, School of Medicine, Wright State University, Dayton, OH, United States

**Keywords:** thiazides, hyperglycemia, metabolic syndrome, loop diuretics, insulin, overweight, hypertension, diabetes

## Abstract

Thiazides, thiazide-like and loop diuretics are commonly prescribed to manage hypertension and heart failure. The main mechanism of action of these diuretics involve inhibition of Na^+^ reabsorption in the kidneys, leading to increased urine production. While effective, diuretics, particularly hydrochlorothiazide, have been linked to altered glucose metabolism and other metabolic issues. These disruptions in fuel homeostasis are not clearly related to their primary action of fluid management, raising concerns for patients with metabolic syndrome, in which high blood pressure coexists with obesity, insulin resistance, glucose intolerance and dyslipidemia. In this review, we conducted an extensive examination of existing literature on these classes of diuretics, covering publications from the late 1950s to the present. Our objective was to investigate the origins, development and current understanding of the widely recognized association between the use of diuretics in general and their potential negative impact on glucose homeostasis. We focused on the clinical and experimental evidence of the most commonly prescribed diuretics: hydrochlorothiazide, chlorthalidone, bumetanide and furosemide. On one hand, the clinical evidence supports the hypothesis that the metabolic effects on glucose homeostasis are primarily linked to hydrochlorothiazide, with little, if any impact observed in other diuretics. In addition, these metabolic effects do not appear to be related to their diuretic action or intended pharmacological targets, raising concerns about the long-term metabolic impact of specific diuretics, particularly in vulnerable populations, including those with metabolic syndrome. On the other hand, the experimental evidence using animal models suggest variable effects of diuretics in insulin secretion and general glucose metabolism. Although the mechanisms involved are not clearly understood, further research is needed to uncover the molecular mechanisms by which certain diuretics disrupt fuel metabolism and contribute to metabolic disturbances.

## 1 Introduction

The common belief that thiazides (including thiazide-like diuretics) and loop diuretics impair glucose metabolism is viewed quite differently by clinicians and scientists. Some consider it a serious concern, while others see it as clinically insignificant (Zhang and Zhao, 2016; Hall et al., 2020), especially in patients with hypertension and coexisting conditions where blood pressure control is the primary, sometimes the only goal (Ramsay et al., 1994; Liang et al., 2017; Hall et al., 2020). In fact, many of the clinical trials from 1966 to 2004 focused solely on hypertension (Carter and Basile, 2005; Zillich et al., 2006) often overlooking coexisting metabolic complications such as those seen in metabolic syndrome (MetS). This condition is defined in hypertensive individuals with a constellation of interconnected metabolic abnormalities significantly increasing the risk of type 2 diabetes (T2D), heart disease and stroke (Samson and Garber, 2014). Indeed, MetS, prevalent among obese individuals, often manifests with glucose intolerance, insulin resistance and dyslipidemia (i.e., hypertriglyceridemia and hypercholesterolemia) (Cornier et al., 2008). As people age and gain weight, the prevalence of MetS also rises, exacerbating non-alcoholic fatty liver disease (NAFLD), recently renamed as metabolic dysfunction-associated fatty liver disease (MAFLD) (Eslam et al., 2020) and hypertension (Collaborators et al., 2017; Moore et al., 2017; Godoy-Matos et al., 2020). In fact, the relationship between hypertension and MetS is complex and bidirectional, with hypertension amplifying the risk of adverse health outcomes when combined with other MetS components (Haffner et al., 1992; Liese et al., 1997; Han et al., 2002). For example, abdominal obesity may contribute to insulin resistance and inflammation, aggravating hypertension, while insulin resistance may directly impact blood vessel function, further worsening hypertension (Kawai et al., 2021). In addition, hypertension worsens insulin resistance and disrupts glucose and lipid metabolism, which increases the risk of cardiovascular diseases and T2D in subjects with MetS (Arnlov et al., 2005; Hu and Stampfer, 2005). Consequently, these pathophysiological interconnections pose a significant clinical challenge when treating hypertensive patients with diuretics (Lassen and Jespersen, 2011).

Surprisingly, despite the extensive literature and the impressive research output over the last 75 years, our understanding of the physiopathological mechanisms underlying diuretic-induced metabolic abnormalities remains notably inadequate. Although some studies have proposed a link between hydrochlorothiazide-induced hypokalemia and elevated blood glucose levels (Carter and Basile, 2005), the causal mechanisms whereby some diuretics were associated with hyperglycemia or glucose intolerance (Zillich et al., 2006; Mukete and Rosendorff, 2013; Scheen, 2018) are unclear and hotly debated, in part due to inconsistent findings (Brown et al., 2015; Hall et al., 2020) and the main focus on hypokalemia as the primary electrolyte imbalance associated with diuretics. However, diuretic-induced sodium depletion may also play an underrecognized role in glucose homeostasis. Sodium is essential not only for the function of sodium-glucose cotransporters (SGLTs) in renal glucose reabsorption (Wright et al., 2007) but also for insulin secretion (Ernst et al., 2009; Nita et al., 2014) and insulin action, indirectly via activation of the renin-angiotensin-aldosterone system (Garg et al., 2011; Zhou et al., 2012). Therefore, both chronic and acute sodium depletion, whether induced by diuretics or other medications, such as antidiabetic SGLTs inhibitors (Ansary et al., 2019; Koh et al., 2023), may contribute to the worsening of metabolic disturbances. Moreover, certain metabolic effects of diuretics observed in animal models or humans, such as glucose intolerance (Zatuchni and Kordasz, 1961; Amery et al., 1978; Giugliano et al., 1980b;Sandstrom, 1988;Sandstrom and Sehlin, 1988d; Sandstrom and Sehlin, 1988b; Kempler et al., 1990;Sandstrom et al., 1993; Lopez et al., 1996; Brown et al., 2015;Brown et al., 2016) and insulin resistance (Bakris et al., 2006; Nathan et al., 2007; Sarafidis et al., 2007; Dronavalli and Bakris, 2008) suggest that diuretics exert effects beyond the kidneys. These findings challenge the assumption that diuretics act solely through renal mechanisms and highlight the need for further investigation into their systemic metabolic consequences.

Growing evidence suggest that thiazides, thiazide-like and loop diuretics may have clinically significant extra-renal effects. Advancements in next-generation sequencing and protein expression profiling have demonstrated that the renal targets of thiazides, i.e., Na^+^Cl^−^ cotransporter (NCC, encoded by *SLC12A3*) and that of bumetanide/furosemide, i.e., Na^+^K^+^2Cl^–^ cotransporter-2 (NKCC2, encoded by *SLC12A1*) are expressed in different tissues and cells, albeit at lower levels (Di Fulvio and Alvarez-Leefmans, 2009). For instance, NKCC2 has been found in insulin-secreting β-cells (Alshahrani et al., 2012), distal colonic epithelia (Zhu et al., 2011) or neurons of the hypothalamus (Konopacka et al., 2015), whereas NCC was detected in endothelial and smooth muscle cells, heart, lung and liver (Wang et al., 2015), adipocytes (Zhang et al., 2022a), and β-cells as well (Zhang et al., 2022b). In addition to that, diuretics may have “non-specific” yet metabolically relevant targets, a phenomenon that has been known from quite some time. For instance, furosemide can inhibit metabolic pathways modulated by several enzymes including UDP-glucuronyltransferases (Sorgel et al., 1980), 11β-hydroxysteroid dehydrogenases (Escher et al., 1995; Fuster et al., 1998), glucose-6-phosphate dehydrogenase, 6-phosphogluconate dehydrogenase and glutathione reductase (Adem and Ciftci, 2016). Similarly, hydrochlorothiazide and bumetanide can inhibit carbonic anhydrase Vb (Kucharczyk et al., 2023) and X (Malebari et al., 2020), respectively, whereas loop diuretics can interfere with signaling mediated by GABA_A_ receptors (Korpi and Luddens, 1997; Thompson et al., 1999) and that of G protein-coupled receptor 35 (Yang et al., 2012). Moreover, bumetanide is well known to inhibit NKCC1, *i.e.*, the *ubiquitous* Na^+^K^+^2Cl^–^cotransporter (Palfrey and Leung, 1993; Hannaert et al., 2002), while furosemide affects multiple K^+^Cl^−^transporters and NKCCs (at higher concentrations) (Haas and McManus, 1983;Popowicz and Simmons, 1988;Hegde and Palfrey, 1992; Lykke et al., 2015), which are unevenly distributed throughout tissues (Adragna et al., 2004; Zhang et al., 2023). Therefore, recognizing that diuretics have diverse pharmacodynamic properties (Wargo and Banta, 2009) and that their pharmacological effects can in turn vary based on many factors including age, gender or ethnicity (Andreasen et al., 1984; Chaudhry et al., 1984; Chun et al., 2008; Conde-Martel et al., 2024), along with the usually disregarded yet significant role of the kidneys in glucose production (Stumvoll et al., 1999; Mather and Pollock, 2011; Alsahli and Gerich, 2017; Legouis et al., 2022; Daza-Arnedo et al., 2023), may reduce bias when evaluating their metabolic effects.

In the next sections, we will briefly introduce thiazides, thiazide-like and loop diuretics from a historic perspective to illustrate how the success of one specific class of these diuretics in treating hypertension overshadowed their undesired metabolic effects and reduced our curiosity to study them to better understand their basic and clinical pharmacology. We will then revisit elemental concepts related to glucose homeostasis to refresh the intricacies of their regulation and identify potential points for future research while focusing on the available evidence relating the use of diuretics with altered fuel homeostasis within the context of the MetS.

## 2 Diuretics: brief historic perspectives

### 2.1 Thiazides and thiazide-like diuretics

Hydrochlorothiazide and chlorthalidone, a benzothiadiazide and a thiazide-like diuretic, respectively were introduced during 1957-59 and the former quickly became a mainstay in the treatment of hypertension. Its popularity at the time stemmed from its perceived clinical (anti-hypertensive) effectiveness, low cost and apparently better safety profiles compared to earlier diuretics (Au and Raisz, 1960; Conway and Lauwers, 1961; Laragh, 1962; Mizgala, 1965). Indeed, hydrochlorothiazide gained widespread popularity following a controlled trial for the management of hypertension published in 1970 (Author Anonymous, 1970). Importantly, over the period spanning the 1960s and 1970s, hydrochlorothiazide underwent continuous examination in many clinical trials, predominantly focused on controlling hypertension (Collins et al., 1990; Antonietta et al., 2022). Almost 20 years after its discovery, a pharmacokinetically dissimilar thiazide-like sulfonamide derivative of hydrochlorothiazide, i.e., chlorthalidone, emerged in the clinic (Riess et al., 1977; Chen and Chiou, 1992). Surprisingly, its efficacy in managing hypertension was first evaluated in 1979 (Author Anonymous, 1979) and several studies, decades later, consistently suggested that this and other thiazide-like diuretics may have a more favorable clinical profile than hydrochlorothiazide (Harrower et al., 1985; Ernst et al., 2006; Chalmers and Arima, 2010; Dorsch et al., 2011; Tziomalos et al., 2013; Liang et al., 2017; Khenhrani et al., 2023). Yet, hydrochlorothiazide prevailed, and still stands, as one of the most frequently prescribed medications in the United States, with a staggering ∼39 million prescriptions for this drug alone in 2021 (clincalc.com/DrugStats/Drugs/Hydrochlorothiazide).

Although there is little evidence that low doses of hydrochlorothiazide (12.5–25 mg daily) reduce the risk of heart attack, stroke or death (Messerli et al., 2011), higher doses have been proven effective in lowering blood pressure and improving cardiovascular outcomes in patients with hypertension. Early trials on small number of subjects during the late 1950s showed the benefits of higher doses, though they did not consider their metabolic effects (Beyer et al., 1957; Bayliss et al., 1958; Bunn, 1958; Freis et al., 1958; Laragh et al., 1958; Rochelle et al., 1958; Wilkins, 1958; Wilkins et al., 1958; Heinemann et al., 1959; Reinhardt, 1959; Laragh, 1967). As our understanding of hypertension grew in the 1980s and 1990s, hydrochlorothiazide remained a key treatment for hypertension (Wilhelmsen et al., 1981; Hebert et al., 1993; Moser and Hebert, 1996; Savage et al., 1998), even as newer drugs with fewer, if any, metabolic issues (e.g., ACE inhibitors, calcium channel blockers and β-blockers) began to replace it (Officers et al., 2002; Grossman and Messerli, 2006). Meanwhile, chlorthalidone was also effective in treating hypertension, as seen in the large ALLHAT trial (Elliott, 1996), which compared different blood pressure medications. Despite some criticisms of this trial (McInnes, 2003; Hebert et al., 2007), its findings heavily influenced future treatment guidelines, promoting the use of *thiazide* diuretics (Chobanian et al., 2003; Ernst and Moser, 2009). However, these guidelines largely focused on hydrochlorothiazide, not chlorthalidone or other thiazide-like drugs like indapamide (Stafford et al., 2010; Messerli and Bangalore, 2011). At this point, it is important to recognize that the term *thiazide* has often been used loosely to refer to hydrochlorothiazide, chlorthalidone and indapamide, despite their pharmacokinetic and pharmacodynamic differences between them (Kurtz, 2010). Over time, each of these diuretics has inherited the benefits and drawbacks of the most commonly prescribed and studied one, i.e., hydrochlorothiazide.

Indeed, early studies did suggest that (*hydrochloro*)thiazide diuretics might be linked to negative effects on glucose metabolism (Johnston and Cornish, 1959; Goldner et al., 1960; Zatuchni and Kordasz, 1961; Runyan, 1962; Author Anonymous, 1963; Anderson, 1966; Author Anonymous, 1971b; Hollenberg and Mickiewicz, 1989; Pollare et al., 1989; Lithell et al., 1990; Plavinik et al., 1992). However, not all research confirmed these findings, with some studies failing to show any such connections (Cornish et al., 1961; Runyan, 1962; Jackson and Nellen, 1966; Andersen and Persson, 1968; Chaudhury et al., 1968; Healy et al., 1970; Berglund et al., 1986; Grimm et al., 1996; Lakshman et al., 1999). The differences in results may be due to variations in study design, dosages, or the populations studied. At any rate, the metabolic effects of hydrochlorothiazide were considered mild or irrelevant from the standpoint of managing hypertension. More recently, however, research has focused on the use of hydrochlorothiazide, alone or in combination with other drugs, for treating hypertension in specific groups (Omboni et al., 2009), such as the elderly and those with obesity, MetS or T2D (Klauser et al., 1991; Middeke et al., 1997; Reisin et al., 1997; Maitland-van der Zee et al., 2005; Siegel et al., 2008; Cooper-DeHoff et al., 2010; Manrique et al., 2010; Gong et al., 2014; Brown et al., 2016; Huang et al., 2016; Georgianos and Agarwal, 2019). Interestingly, no substantial effects of hydrochlorothiazide or chlorthalidone on plasma insulin were reported in several of these and other trials (Klauser et al., 1991; Plavinik et al., 1992; Price et al., 2013). However, there has been less emphasis on thiazide-like diuretics, despite evidence that both chlorthalidone and indapamide may offer better metabolic outcomes compared to hydrochlorothiazide (Kostis et al., 1997; Black et al., 2008; Karnes et al., 2014; Singh et al., 2018). With apparently few exceptions (Jian-Liang et al., 2004), these drugs can reduce blood pressure with less impact on blood glucose and cholesterol levels (Liang et al., 2017). Despite this, many studies, especially those involving hydrochlorothiazide, have led to the widespread belief that diuretics invariably affect glucose metabolism, regardless of their class or specific characteristics.

### 2.2 Loop-diuretics

The story of loop diuretics began in the early 1960s, when ethacrynic acid was found to increase urine production in both animals and humans (Beyer et al., 1962; Bernstein et al., 1965; Cannon et al., 1965). Ethacrynic acid became the first non-sulfonamide loop diuretic used in clinical settings, leading to the development of more powerful loop diuretics. In the mid-to-late 1960s, furosemide was synthetized and quickly gained popularity due to its strong diuretic effects, fast action and effectiveness, especially in treating heart failure, hypertension, edema and kidney failure (Ingram, 1964; Godwin and Gunton, 1965; Laragh et al., 1966; Stason et al., 1966; Davidov et al., 1967; Earley, 1967; Walker, 1967; Joynt and Morrin, 1968; Kirkendall and Stein, 1968; Cannon and Kilcoyne, 1969; Shanoff, 1969). By the 1970s, furosemide became one of the most commonly prescribed diuretics, with fewer undesired effects compared to earlier diuretics, including hydrochlorothiazide (Feit, 1971; Wertheimer et al., 1971; Cannon, 1972; Valmin and Hansen, 1975; Mahabir and Bacchus, 1976; Finnerty et al., 1977; Araoye et al., 1978; Coodley et al., 1979; Dettelbach and Bennett, 1979). Other loop diuretics with better bioavailability and longer-lasting effects, such as bumetanide, torsemide, azosemide and piretanide, were introduced around this time as well (Asbury et al., 1972; Murdoch and Auld, 1975; Hettiarachchi et al., 1977; Jayakumar and Puschett, 1977; Benet, 1979; Brater et al., 1979; Konecke, 1981; Whelton, 1981; Stroobandt et al., 1982; Halstenson and Matzke, 1983; McNabb et al., 1984; Ward and Heel, 1984; Clissold and Brogden, 1985; Car et al., 1988). Many of these diuretics are still in use today (Blose et al., 1995; Bagshaw et al., 2007; Carone et al., 2016; Mentz et al., 2016; Rahhal et al., 2019; Singh et al., 2023). Even after 40 years, loop diuretics remain a key treatment for conditions involving excess fluid retention, as supported by ongoing clinical trials (Blake, 1990; Kissling and Pickworth, 2014; Ozieranski et al., 2019; Eid et al., 2021; Verbrugge and Menon, 2022; Greene et al., 2023; Mentz et al., 2023; Cuthbert and Clark, 2024; Kapelios et al., 2024; Krim et al., 2024).

However, most clinical trials on loop diuretics over the past 50 years have primarily and understandably focused on how they affect edema and electrolyte balance, rather than their potential impact on glucose metabolism. As a result, there is limited evidence linking loop diuretics to metabolic issues, especially compared to hydrochlorothiazide. However, early on, loop diuretics seemed to inherit the perceived metabolic effects of hydrochlorothiazide (Toivonen and Mustala, 1966). This concern may have originated from a 1959 study that first raised the possibility of diuretics affecting glucose metabolism (Freis and Finnerty, 1959) based on the effects of hydrochlorothiazide. Although there are few direct studies connecting loop diuretics (such as furosemide) to metabolic problems (Lavender and McGill, 1974; Tasker and Mitchell-Heggs, 1976; Khaleeli and Wyman, 1978), isolated cases of glucose intolerance or diabetes in patients using furosemide have been reported. Nevertheless, one study in 1966 found that furosemide had little effect on glucose tolerance over 3 months in both healthy people and those with hypertension (Jackson and Nellen, 1966). Another study suggested that ethacrynic acid also had minimal effects on glucose levels in mildly hypertensive patients (Andersen and Persson, 1968). Yet, later reports documented some cases of glucose intolerance associated with furosemide (Coni et al., 1974; Cowley and Elkeles, 1978; Kobayakawa et al., 2003). On the other hand, short-term studies showed no significant impact on blood sugar levels from either furosemide or bumetanide in both healthy individuals and patients with T2D (Asbury et al., 1972; Kaldor et al., 1975). Similarly, studies in 1980 indicated that neither diuretic had a significant effect on insulin or glucagon secretion (Giugliano et al., 1980b; Luyckx et al., 1980), though furosemide did slightly alter insulin and glucagon responses without affecting glycemia (Giugliano et al., 1980a). In 1981, a study found that bumetanide even improved glucose tolerance, but furosemide did not (Robinson et al., 1981). Further research indicated that, unlike hydrochlorothiazide, bumetanide had no significant effect on insulin or other hormone levels in dog pancreas models (Hermansen et al., 1985). The introduction of piretanide in the 1980s also did not consistently affect glucose tolerance or insulin levels, though both piretanide and furosemide were linked to changes in cholesterol levels in hypertensive patients (Valimaki et al., 1983; Weidmann et al., 1983; Campbell et al., 1998). However, later studies did not confirm these findings consistently (Harno et al., 1985; Chaudhuri and Catania, 1988; Weidmann et al., 1993; Lind et al., 1995; van der Heijden et al., 1998). In fact, piretanide (Harno et al., 1985) and likely bumetanide (Harno et al., 1985) increased insulin secretion in humans.

Therefore, overall, it appears that the *“diabetogenic”* risks commonly associated with diuretics are more strongly linked to hydrochlorothiazide (Padwal and Laupacis, 2004; Stump et al., 2006) than other classes of diuretic or anti-hypertensive medications. Although “meta-analysis (97 comparisons across 95 trials) demonstrated a statistically significant but clinically unimportant increase in FPG [fasting plasma glucose]” (Hall et al., 2020), the impact of any diuretic on glucose homeostasis seems to depend on several factors, including the type of diuretic used and the specific metabolic context on which these diuretics are being studied (Grossman et al., 2011).

## 3 Effects of diuretics on glucose homeostasis

The common belief that diuretics negatively affect fuel balance in humans lacks strong experimental support, particularly for thiazide-like and loop diuretics. Nonetheless, we will focus on reviewing experimental evidence, mostly from animal studies, to better understand the potential effects of hydrochlorothiazide and loop diuretics on key processes involved in glucose regulation. This includes their impact on insulin secretion and the production and use of glucose in the liver and kidneys.

**FIGURE 1:**
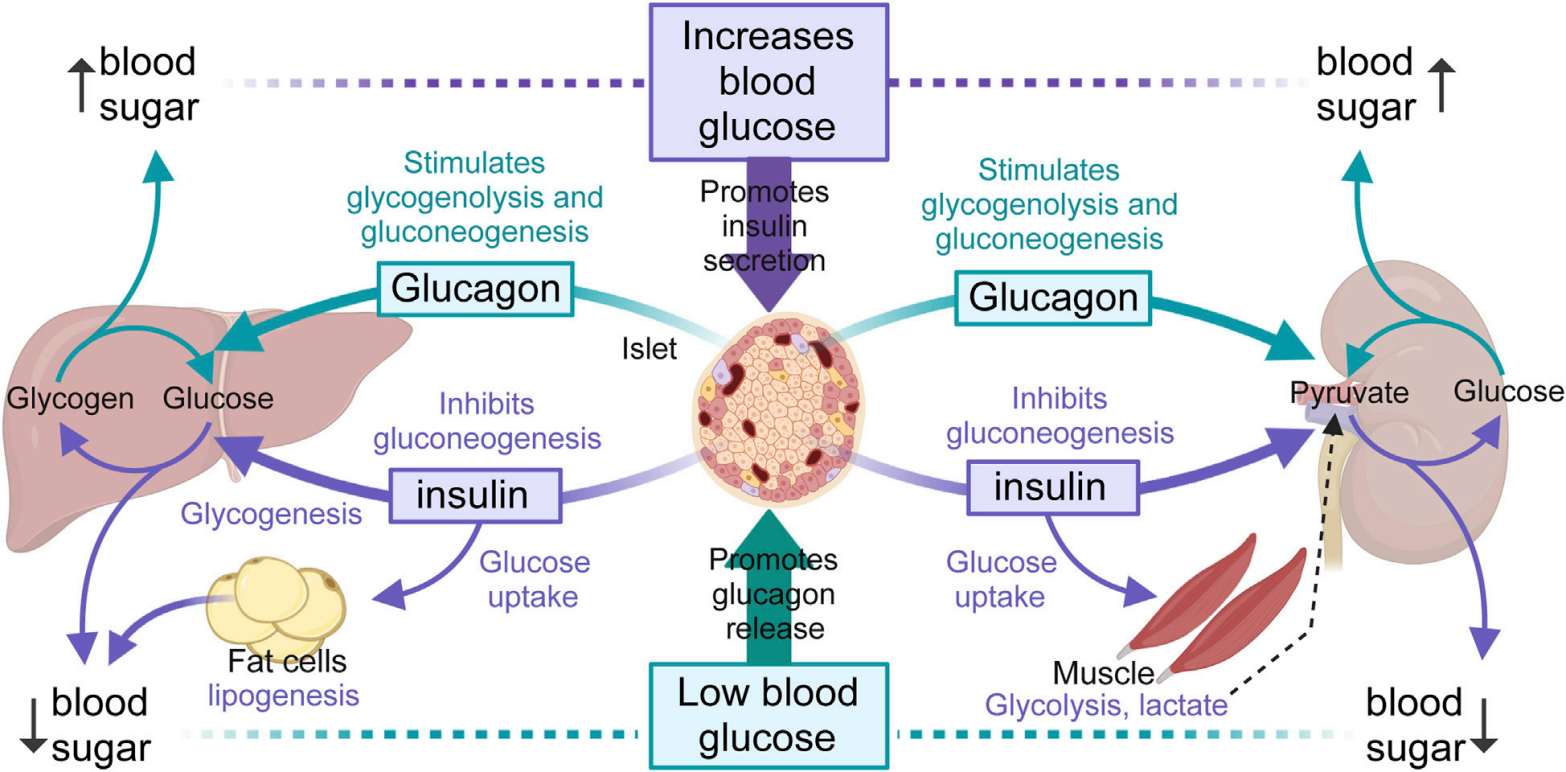
Overview of blood glucose regulation. The liver, muscles and kidneys are major modulators of blood glucose levels by releasing glucose through glycogenolysis (liver, muscle) and gluconeogenesis (liver and kidneys), in turn orchestrated by insulin (purple arrows) and glucagon (green arrows) secreted by β- and α-cells of the pancreatic islet, respectively. Glycogenolysis breaks down stored glycogen into glucose-6-phosphate, then free glucose after dephosphorylation, while gluconeogenesis forms glucose-6-phosphate from various non-hydrocarbon precursors (e.g., pyruvate, lactate, glycerol, glutamine). Only the liver, kidneys and small intestines (not represented) can release glucose from glucose-6-phosphate due to the presence of glucose-6-phosphatase activity. Hepatic glycogen breakdown releases glucose, while muscle glycogen breakdown releases lactate, a substrate that can be converted back into glucose by the liver and kidneys after conversion to pyruvate. The kidneys use glucose mainly in the renal medulla and release it from the renal cortex, due to enzyme differences alone the nephron. Renal medulla cells, like neurons, can accumulate glycogen but cannot release glucose. Renal cortex cells can produce and release glucose but cannot synthesize glycogen. In adipocytes, insulin promotes the uptake of glucose and its transformation into fat.

Insulin secreted from β-cells of the islets of Langerhans in the pancreas promotes the uptake of glucose from the blood into muscle and other insulin-sensitive tissues for immediate use (glycolysis) or fat storage (lipogenesis). In contrast, glucagon secreted by α-cells of the islet, has the opposite effect of insulin. When glycemia is low, such as during fasting or between meals, glucagon promotes the hepatic break-down of stored glycogen (glycogenolysis) into glucose for release into the bloodstream, or the renal synthesis of glucose from non-carbohydrate sources (Jiang and Zhang, 2003; Mutel et al., 2011; Bankir et al., 2016). Importantly, the liver and the kidneys, and to a much lesser extent the small intestine can produce glucose from amino acids and glycerol, through a process called *de novo* gluconeogenesis. This ensures a steady supply of glucose for organs and tissues, especially during long periods of fasting or prolonged exercise. In the case of insulin-sensitive tissues, such as the muscles and adipose tissue (Booth et al., 2016; Merz and Thurmond, 2020), when insulin binds to its receptors, glucose transporters (*e.g.*, GLUT4) translocate to the cell membrane allowing glucose to enter the cell, where it can be used for energy during exercise or stored as glycogen (muscle) for future use. In adipose tissue, fat cells store energy in the form of triglycerides. On one hand, fatty acids produced from triglycerides by lipolysis can be used as an energy source by many tissues, including muscle cells (Hargreaves and Spriet, 2020). On the other hand, glycerol, also produced from triglycerides by lipolysis, can be converted into glucose through gluconeogenesis in the liver, providing an additional source of glucose during fasting or periods of increased energy demand (Han et al., 2016). These concepts, outlined in Figure 1, are relevant for our discussion; as insulin secretion, the glycolytic and/or lipolytic potential of tissues, gluconeogenesis and likely most aspects of glucose and energy homeostasis have been found defective and implicated in the pathogenesis and/or progression of hypertension and MetS (Katsimardou et al., 2020).

### 3.1 Effects of diuretics on insulin secretion: the evidence

**FIGURE 2:**
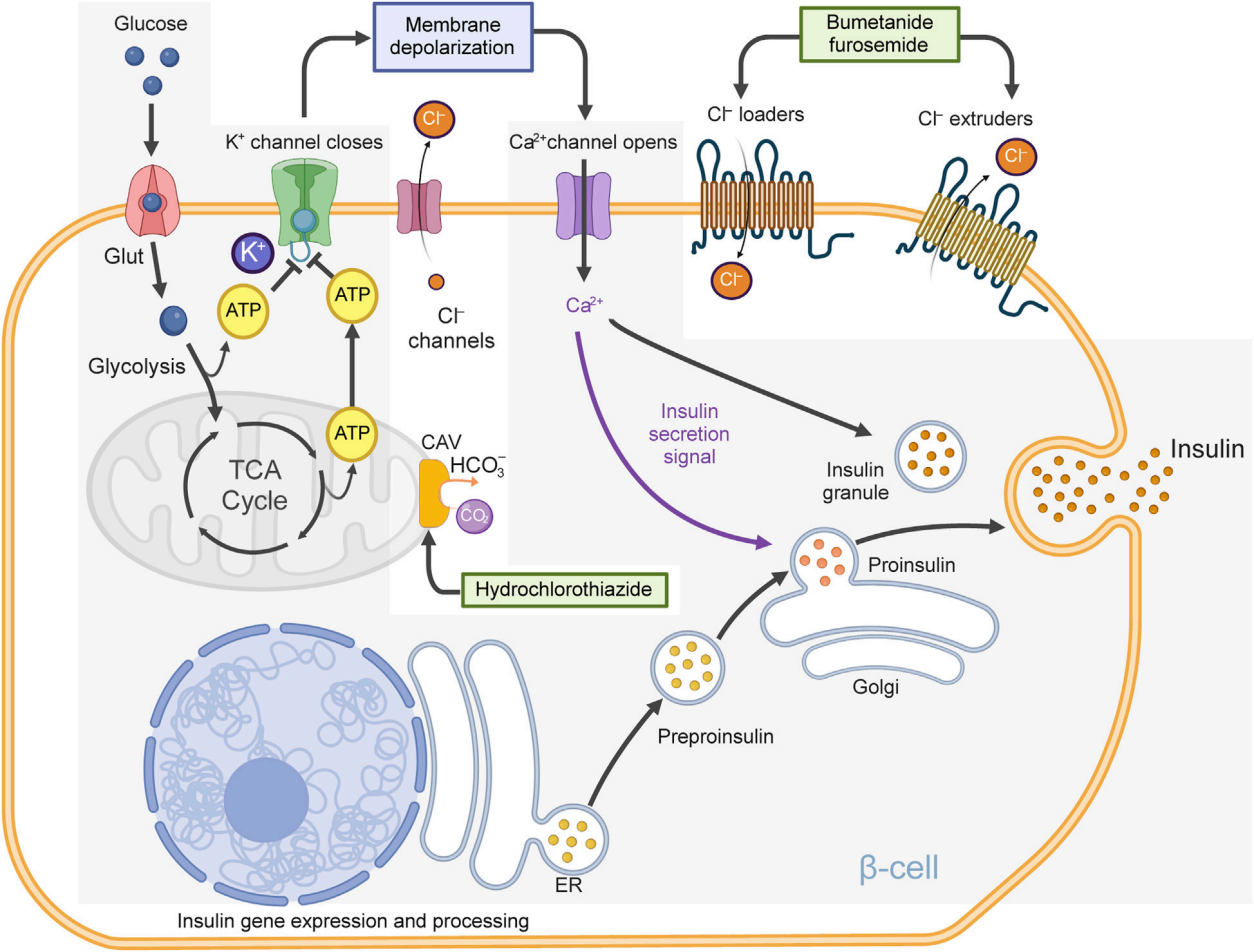
Oversimplified model of insulin secretion. Described is a β-cell containing glucose transporters (Glut), K_ATP_-channels, voltage-gated Ca^2+^ channels, bumetanide-sensitive Cl^−^ loaders (e.g., *NKCC2, NKCC1*), furosemide-sensitive Cl^−^ extruders (e.g., *KCC1, KCC2, KCC3, KCC4*) and Cl^−^ channels [e.g., volume-regulated anion channels, (*VRAC*), Ca^2+^ activated Cl^−^ channels (*ANO1*) and others]. Note that Cl^−^ loaders and extruders help maintain the intracellular Cl^−^ concentration above thermodynamic equilibrium, making possible the electrogenic exiting of Cl^−^ ions, when Cl^−^ channels are opened, contributing to plasma membrane depolarization. When glucose is transported into the β-cell, it undergoes glycolysis, generating ATP and metabolites that affect cellular osmolarity and cell volume. ATP closes K_ATP_-channels, reducing K^+^ permeability and causing plasma membrane depolarization. Metabolites and Ca^2+^ open Cl^−^ channels triggering inward Cl^−^ currents (Cl^−^ exits the cell). Many Cl^−^ channels likely contribute to these currents which together with reduced K^+^ permeability are responsible for the activation of voltage-gated Ca^2+^ channels, thus leading to Ca^2+^ influx, action potentials, electrical activity and insulin release. Note: hydrochlorothiazide can inhibit mitochondrial carbonic anhydrase Vb (CAV), which limits the supply of HCO_3_^−^ to pyruvate carboxylase (and other carboxylases) reducing the biosynthesis of oxaloacetate, an intermediary of the tricarboxylic acid (TCA) cycle potentially reducing ATP and contributing to reduced K_ATP_-channel closure. The *consensus model* of insulin secretion is greyed.

The process by which nutrients, particularly glucose, trigger insulin secretion from islet β-cells is complex and involves many signals (Di Fulvio et al., 2014). However, medical textbooks often oversimplify this process. Typically, the consensus mechanism is described as follows (see Figure 2): When glucose enters β-cells, it undergoes glycolysis, which raises intracellular ATP levels. This increase in ATP closes ATP-sensitive K^+^ channels (K_ATP_ channels), causing depolarization of the cell membrane. As a result, voltage-gated Ca^2+^ channels open, allowing Ca^2+^ to flow into the cell. The influx of Ca^2+^ triggers the release of insulin from the β-cells into the bloodstream. While this mechanism is important, it is incomplete (Henquin et al., 2009; Merrins and Kibbey, 2024). Indeed, Cl^−^ channels and Cl^−^ transporters also help regulate β-cell membrane potential and excitability, both crucial for insulin release (Best et al., 2010). In fact, recent studies have clearly defined the roles of some of these Cl^−^ channels in islet physiology (Crutzen et al., 2016; Kang et al., 2018; Stuhlmann et al., 2018; Di Fulvio et al., 2020) and importantly, some Cl^−^ transporters help maintain the intracellular Cl^−^ concentration ([Cl^−^]_i_) above its predicted thermodynamic equilibrium, facilitating the movement of Cl^−^out of the cell and through Cl^−^ channels in an electrogenic manner. Notably, some of these Cl^−^ transporters in β-cells can be directly targeted by thiazide and loop diuretics (Di Fulvio and Aguilar-Bryan, 2019). In fact, hydrochlorothiazide (Hoskins and Jackson, 1978; Sandstrom et al., 1993; Kucharczyk et al., 2023), trichlormethiazide (Seltzer and Allen, 1969), hydroflumethiazide (Hermansen et al., 1985), bumetanide (Hermansen et al., 1985; Sandstrom, 1990), furosemide (Aynsley-Green and Alberti, 1973; Hermansen et al., 1986; Sandstrom and Sehlin, 1988c; Eberhardson et al., 2000) and indapamide (Hermansen et al., 1986) can all influence insulin secretory responses *in vitro* and *in vivo* in animal models. In addition, hydrochlorothiazide, bumetanide and furosemide were also consistently linked to altered blood glucose and impaired glucose tolerance in a variety of animal models (Foy, 1967; Weller and Borondy, 1967; Foy and Furman, 1969; Foy and Furman, 1971; Foy and Furman, 1972; Hoskins and Jackson, 1978;Papaccio and Esposito, 1987; Sandstrom, 1988; Sandstrom and Sehlin, 1988d; Sandstrom and Sehlin, 1988b; Ray et al., 1993; Sandstrom et al., 1993). Therefore, these data support the hypothesis that the metabolic effects associated with the use of thiazide, thiazide-like, loop-diuretics are related, at least in part, to direct or indirect effects on islet β-cell secretory function.

#### 3.1.1 Effects of thiazides on insulin secretion: the mechanisms

At first sight, the reported influence of hydrochlorothiazide on insulin secretion from rodent islets *in vitro* (Malaisse and Malaisse-Legae, 1968; Sandstrom et al., 1993) might now seem related to inhibition of NCC. However, the transcript levels of *SLC12A3* were consistently very low or undetectable in both human and rodent islets, as determined by traditional methods (Zhang et al., 2022b) or advanced transcriptome profiling (Riahi et al., 2018; Jaafar et al., 2019; Chen et al., 2022). Moreover, hydrochlorothiazide have been shown to diminish insulin secretion from islets of obese mice by reducing Ca^2+^ influx rather than altering [Cl^−^]_i_, K^+^ or Cl^−^fluxes (Sandstrom et al., 1993). Therefore, the potential adverse effects possibly induced by hydrochlorothiazide on the islet secretory function might be influenced by targets other than NCC. Indeed, it is known that hydrochlorothiazide can target several ion transporters and enzymes including *SLC4A8*, a Na^+^-dependent Cl^−^/HCO_3_^−^ exchanger (NDCBE), *SLC26A4*, a Na^+^-independent Cl^−^/HCO_3_^−^ exchanger (Pendrin) and carbonic anhydrases (Pickkers et al., 1999; Leviel et al., 2010; Sinke et al., 2014), all of which were shown to play roles in insulin secretion (Parkkila et al., 1998; Sener et al., 2007). In fact, recent data suggest that hydrochlorothiazide may inhibit insulin secretion from normal mouse islets by blocking the activity of mitochondrial carbonic anhydrase Vb (Kucharczyk et al., 2023) (see Figure 2). Importantly, this enzyme provides HCO_3_^−^ ions to different enzymes that participate in intermediary metabolism including pyruvate carboxylase (anaplerosis, gluconeogenesis), propionyl-CoA carboxylase, 3-methylcrotonyl-CoA carboxylase (branched chain amino acids catabolism) and carbamoylphosphate synthase 1 (urea cycle). Therefore, it is plausible that hydrochlorothiazide, by targeting carbonic anhydrases and other enzymes, may have wider metabolic effects than predicted, at least in animal models.

At any rate, the long-term effects of hydrochlorothiazide treatment on essential metabolic parameters such as body weight, body composition and dynamic evaluations of glucose homeostasis and metabolomics have not yet been conducted. Furthermore, the long-term role of renal NCC in the regulation and/or maintenance of glycemia also remains unknown. This is relevant, as thiazides in general have been proposed to promote metabolic dysregulation by inhibiting insulin secretory responses to nutrients through their hypokalemic effects (Zillich et al., 2006), and perhaps by direct effects on renal gluconeogenesis (Fulgraff et al., 1972). Moreover, the current hypothesis that hydrochlorothiazide may worsen glucose homeostasis through mechanisms related to insulin secretion has been recently challenged. Indeed, islets from young mice lacking NCC or NDCBE (NCC^KO^ or NDCBE^KO^, respectively) exhibited normal secretory responses to glucose (Kucharczyk et al., 2023). Yet, hydrochlorothiazide triggered acute glucose intolerance in these mice. Hence, this diuretic can have metabolic effects independently of both transporters and by mechanisms unrelated to direct effects on islet NCC o NDCBE.

Nevertheless, a recent study has confirmed the presence of NCC in some but not all insulin-positive β-cells of both human and rodent islets (Zhang et al., 2022b). In these contexts, it was proposed that NCC may act as a receptor for interleukin 18 (IL-18), potentially collaborating with receptors for the incretin glucagon-like peptide 1 (GLP-1) to enhance β-cell mass and help maintain glucose homeostasis (Zhang et al., 2022b). Although it is unknown if the ion transport activity of NCC is required to interact with IL-18 (Wang et al., 2015), the potential functional/molecular interplay between NCC and GLP-1 receptors implies a permissive role for the former in the prandial islet secretory response to incretins. Notably, the insulinotropic effect of GLP-1 was lost in islets of NCC^KO^ mice (Zhang et al., 2022b). Nevertheless, it remains unknown whether hydrochlorothiazide or thiazide-like diuretics reduce GLP-1 responses, glucose tolerance, energy intake behavior and feeding patterns in the long term. This constitutes an interesting hypothesis to test given that mice deficient in IL-18 signaling are insulin resistant, hyperphagic and obese (Netea et al., 2006; Zorrilla et al., 2007; Pazos et al., 2015). Further, some of IL-18 effects may be mediated by NCC (Wang et al., 2015; Zhang et al., 2022a; Zhang et al., 2022b) and potentially sensitive to thiazides and thiazide-like diuretics.

Like NCC^KO^ islets, those from mice lacking NCC exclusively in β-cells (NCC^βKO^) showed preserved glucose-stimulated insulin secretion. However, these mice had reduced β-cell mass and enhanced islet inflammation under high fat diet (HFD) conditions (Zhang et al., 2022b). Therefore, it has become clear that β-cells can release insulin without relying on NCC, especially when triggered by glucose, although this may not be the case for other stimuli, including that elicited by GLP-1. Further, the data also imply a role for NCC in inflammatory processes, which may be of clinical relevance given the relationship that exists between low grade local tissue inflammation, obesity and the progression of MetS (Aronson et al., 2004; Grundy et al., 2005; Haffner, 2006). Although hydrochlorothiazide did impair glucose tolerance in normal mice through mechanisms related to β-cell insulin secretion, but independent of NCC (Kucharczyk et al., 2023), NCC^βKO^ mice did not show reduced insulin responses to exogenous glucose. In fact, NCC^βKO^ and NCC^KO^ mice were normotolerant to glucose (Zhang et al., 2022b; Kucharczyk et al., 2023). Consequently, when considered collectively, these data suggest that hydrochlorothiazide could potentially induce glucose intolerance, particularly in mice models, through various mechanisms including those partially associated with β-cell function and mass, those related to intermediary metabolism, alongside others yet to be uncovered.

At any rate, the long-term role of NCC either as an ion transporter sensitive to thiazides, as an IL-18 receptor or as a potential partner for GLP-1 receptors in β-cells or in any capacity in metabolically active tissue awaits exploration, particularly within the context of obesity, the most prevalent component of MetS. Along these lines, HFD-fed NCC^βKO^ mice showed exacerbated body weight gain, glucose intolerance and insulin resistance relative to chow fed mice (Zhang et al., 2022a; Zhang et al., 2022b). Therefore, it is possible that β-cell NCC may play a protective role against overnutrition and metabolic dysregulation. While it is uncertain whether these alterations also involve modified incretin responses, within the framework of obesity, MetS and the use of hydrochlorothiazide for treating hypertension associated with these conditions, these findings suggest an intriguing hypothesis: that overweight or overnutrition might amplify the metabolic effects of these diuretics by inhibiting NCC and/or other targets in β-cells and in metabolically active tissues but independently of insulin secretion.

#### 3.1.2 Effects of loop diuretics on insulin secretion: the mechanisms

When considering the potential metabolic effects of loop diuretics, a similar contextual line of thought as that conveyed for hydrochlorothiazide can be pragmatic. Certainly, several “extrarenal hypotheses” have been proposed over the years to better understand some observed metabolic effects of loop diuretics, mostly bumetanide and furosemide, in humans and animal models. For instance, it has been known for quite some time that these two diuretics may directly impair insulin secretion from islets *in vitro* and deteriorate glucose tolerance in mice (Sandstrom, 1988; Sandstrom and Sehlin, 1988d; Sandstrom and Sehlin, 1988b; Sandstrom, 1990; Sandstrom et al., 1993). Importantly, the demonstrated acute *in vitro* inhibitory effects of low concentrations of bumetanide on islet insulin secretion (Sandstrom, 1990) seem to stem mostly from inhibition of NKCC1, as its exclusive elimination from β-cells precluded the effects of bumetanide (Abdelgawad et al., 2022).

However, experiments using islets of null mice lacking NKCC1 (NKCC1^KO^) gave unexpected results. Contrary to initial expectations, pancreatic islets from 3-4w old NKCC1^KO^ mice showed exaggerated insulin responses to glucose *in vitro* rather than a reduced response (Alshahrani and Di Fulvio, 2012). These data suggest that NKCC1 is dispensable for insulin secretion and that the dependence of insulin secretion on acute inhibition of NKCCs by bumetanide (Best, 2005) or furosemide (Sandstrom and Sehlin, 1988c;a) is rather complex. Along these lines, NKCC1^KO^ mice exhibited exaggerated glucose tolerance (Alshahrani and Di Fulvio, 2012), which is also surprising given the well-known detrimental effects that bumetanide and furosemide have on glucose tolerance in mice (Sandstrom, 1988; Sandstrom and Sehlin, 1988d; Sandstrom and Sehlin, 1988b). Although these results are challenging to reconcile from a metabolic perspective, especially when considering that NKCC1^KO^ null mice display a range of developmental and functional abnormalities (Delpire et al., 1999; Flagella et al., 1999; Evans et al., 2000; Meyer et al., 2002b; Walker et al., 2002; Bradford et al., 2016), recent data from patients harboring inactivating mutations in the *SLC12A2* gene have suggested a potential implication for NKCC1 in intestinal function (Koumangoye et al., 2020) and energy metabolism (Omer et al., 2020). At any rate, the role of NKCC1 in insulin-secreting β-cell function is likely influenced by redundant mechanisms. In addition to NKCC1, islet β-cells express low levels of NKCC2A, a spliced variant of *SLC12A1* (i.e., *SLC12A1v1*) (Alshahrani et al., 2012) exquisitely sensitive to bumetanide but functionally different than NKCC1 (Zeuthen and Macaulay, 2012). In fact, bumetanide did inhibit insulin secretion from NKCC1^KO^ islets and impaired glucose tolerance in NKCC1^KO^ mice (Alshahrani and Di Fulvio, 2012) whereas mice hemizygous for NKCC1 showed improved glucose tolerance associated to increased expression of NKCC2A in islet β-cells (Alshahrani et al., 2015). Therefore, it is plausible that NKCC2 may compensate, at least to some extent, the functional decrease or even absence of islet NKCC1 and play a minor, if any role *per se* in the secretory response. In line with this assumption, *in vitro* insulin responses to glucose from NKCC1-expressing islets but lacking NKCC2A were normal (Kelly et al., 2019). However, NKCC2A^KO^ islets also showed increased expression of KCC2, i.e., a furosemide-sensitive and constitutively active K^+^Cl^−^ cotransporter (Payne, 1997; Williams and Payne, 2004) recently implicated in facilitating insulin secretion (Kursan et al., 2017; Pae and Harper, 2021).

From the previous lines, it has become evident that β-cells possess overlapping, loop diuretic-sensitive mechanisms, which complicates the dissection of the specific role of each of them. Indeed, in addition to NKCC1, many K^+^Cl^−^ cotransporter variants have been found at the mRNA levels in mammalian islets including KCC1, three and four splice variants of KCC2 and KCC3, respectively, and KCC4 (Davies et al., 2004; Kursan et al., 2017). Although these KCC variants are considered sensitive to loop diuretics, but not functionally equivalent (Adragna et al., 2004), our knowledge regarding the roles of these transporters in insulin secretory responses *in vitro* or glucose homeostasis *in vivo* is scant. Mammalian β-cells and islets do have furosemide-sensitive K^+^Cl^−^extrusion mechanisms, which become robust in response to cell swelling (Engstrom et al., 1991). As such, these transporters have been implicated in the quick inhibitory effect that furosemide has on islet insulin secretion *in vitro* (Aynsley-Green and Alberti, 1973;Hermansen et al., 1986;Sandstrom and Sehlin, 1988d;c;a;Eberhardson et al., 2000). Intriguingly, high doses of furosemide stimulated insulin secretion *in vitro* (Sandstrom and Sehlin, 1988c) producing a U-shaped dose-response like that observed with high doses of bumetanide (Sandstrom, 1990). Notably, these effects on islet insulin secretion were paralleled by changes in Cl^−^ and Ca^2+^ fluxes (Sandstrom and Sehlin, 1987; 1988a; Sandstrom, 1990). However, while these experiments did not distinguish which KCC may be involved in the stimulatory effects of high doses of the diuretic, inhibition of β-cell KCC2 with highly selective drugs (Kursan et al., 2017) or its transient siRNA-mediated downregulation in islets (Pae and Harper, 2021) resulted in increased insulin secretion in response to glucose. Yet, the *in vivo* role of β-cell KCC2, or that of KCC1, KCC3 or KCC4 on glucose homeostasis, if any, remain to be explored.

The use of mice lacking NKCC1 specifically in insulin-secreting β-cells (NKCC1^βKO^) has provided some insight into the long-term metabolic effects of the bumetanide-sensitive NKCC1 in insulin secreting cells. For instance, NKCC1^βKO^ mice gradually became overweight, hyperinsulinemic, hyperglycemic, hypertriglyceridemic, glucose intolerant and insulin resistant while developing mild non-alcoholic steatohepatitis and reduced β-cell mass and function, i.e., typical conditions found in MetS (Abdelgawad et al., 2022). Although the precise causal mechanisms underlying the initiation of this phenotype in NKCC1^βKO^ mice remain unresolved, it is evident that fundamental deficiencies in β-cell function and/or mass are pivotal in the development/progression of age-dependent metabolic dysregulation (Hudish et al., 2019). Interestingly, NKCC1^βKO^ mice also showed reduced satiation control to *ad libitum* feeding before developing overweight and a MetS-like phenotype (Rathod et al., 2023), consistent with the hypothesis that islets hormones participate in the control of food/energy intake (Woods et al., 2006). In that regard, it is known that chronic low doses of furosemide and potentially other diuretics can increase long-term energy intake in animal models (National Toxicology, 1989a; National Toxicology, 1989b; Bucher et al., 1990). Therefore, these data raise an intriguing possibility; in addition to provoke diuresis, loop diuretics may indirectly modulate feeding behavior and/or energy balance. However, as it is the case of many drugs in clinical use today, the role of diuretics in the behavioral control of food intake awaits further exploration.

### 3.2 Effects of diuretics on renal glucose production

**FIGURE 3:**
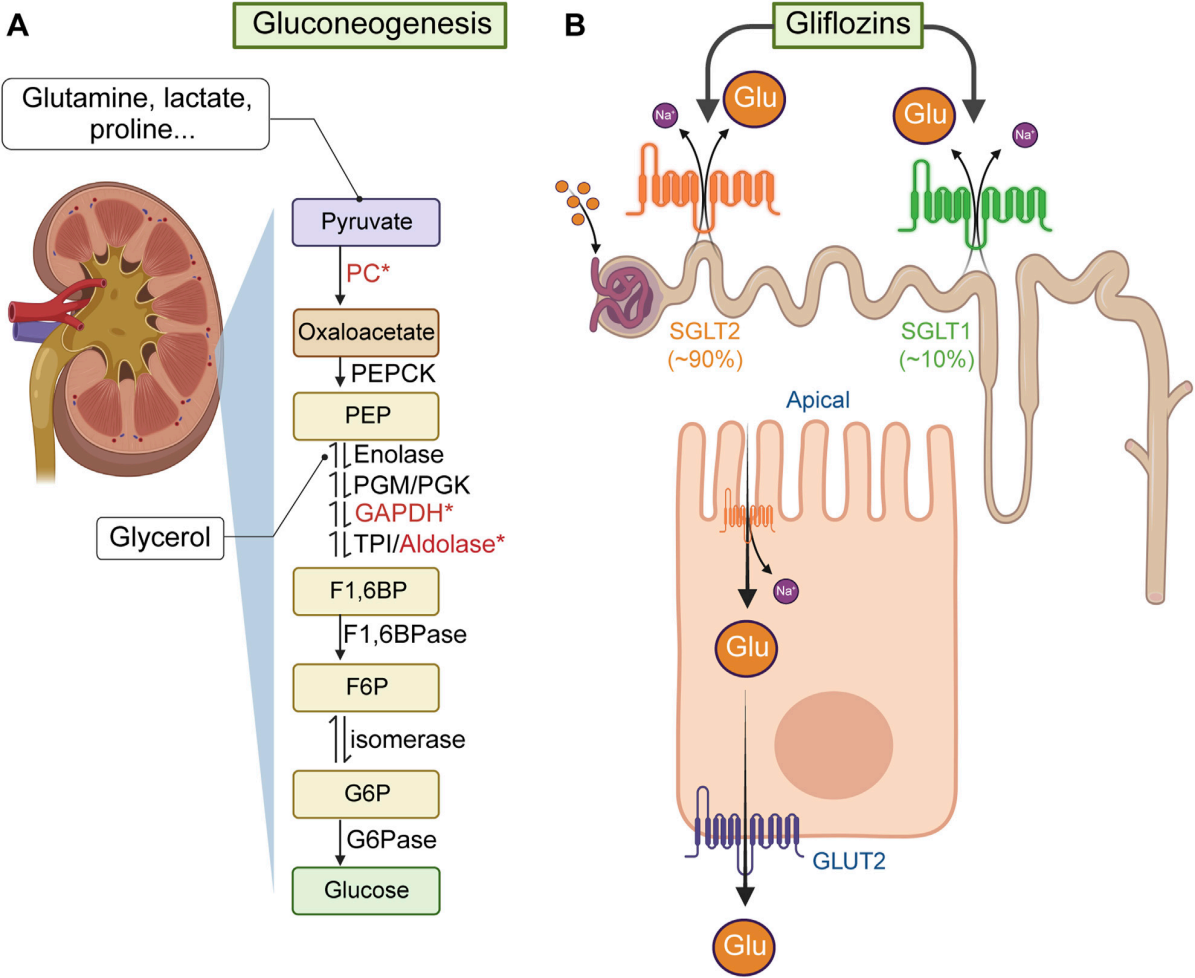
Renal *de novo* gluconeogenesis and glucose reabsorption. **(A)** Renal *de novo* gluconeogenesis is the process by which the kidneys produce glucose from non-carbohydrate sources (e.g., lactate, glycerol, amino acids). This process mainly occurs in the renal cortex and is particularly important during periods of fasting or intense exercise. Lactate or glutamine (from muscle) generate glucose in the kidneys after being transported into renal tubular cells, where they undergo enzymatic reactions to form pyruvate, which then is converted into oxaloacetate via pyruvate carboxylase (PC, which uses HCO_3_^−^ provided by carbonic anhydrases, some of them potentially inhibited by hydrochlorothiazide). Oxaloacetate, through phosphoenolpyruvate carboxykinase (PEPCK) forms phosphonolpyruvate (PEP). Glycerol (from adipocytes), can enter the gluconeogenic process as a precursor of glyceraldehyde-3-phosphate (G3P) by the enzymes glyceraldehyde 3-phosphate dehydrogenase (GAPDH) and triose phosphate isomerase (TPI). G3P combined with dihydroxyacetone-phosphate, via aldolase B, forms fructose-1,6-bisphosphate (F1,6BP). Note: GAPDH was reported inhibited by furosemide and ethacrynic acid, and aldolase B can directly regulate *NKCC2* functional expression. F1,6BP is then dephosphorylated to fructose-6-phosphate via fructose-1,6-bisphosphatase (F1,6BPase) and isomerized to form glucose-6-phosphate. **(B)** Renal glucose reabsorption primarily occurs in the proximal tubule of the nephron, ensuring that glucose is conserved and returned to the bloodstream rather than excreted in urine. This process involves two main types of glucose transporters: *SGLTs* and *GLUTs*. In particular, *SGLT2*, located in the apical side of epithelium of the proximal convoluted tubule uses the Na^+^ gradient to reabsorb ∼90% of glucose from the filtrate back into the cells lining the tubule. The remaining glucose filtered is absorbed by *SGLT1* further down the proximal tubule. Once in the tubular cell, *GLUT2*, located in the basolateral side of the tubular epithelium, transports glucose into the bloodstream.

The kidneys produce and release glucose primarily through gluconeogenesis (Weber, 1961; Schoolwerth et al., 1988) (Figure 3A). In fact, the kidneys contribute ∼50% of the total glucose released into the systemic circulation under fasting conditions (Gerich et al., 2001). Moreover, increased renal glucose production is a possible contributor to the development of hyperglycemia in patients with insulin resistance and MetS (Legouis et al., 2022). Indeed, insulin regulates renal gluconeogenesis by influencing enzyme production or activity associated with the availability of gluconeogenic precursors (Cano, 2001), an influence anticipated to be diminished or impaired in individuals with MetS or obesity-related insulin resistance (Rebelos et al., 2024). Yet, it remains uncertain whether any individual component of MetS, either alone or in combination, affects the gluconeogenic capacity of the kidneys. Much less certain is the potential effects that diuretics may have on renal glucose production.

Nevertheless, studies performed ∼30 years ago have shown that furosemide and ethacrynic acid can inhibit mitochondrial electron transport in renal tissues (Manuel and Weiner, 1976; Orita et al., 1983) and that diuretics, in general, appear to have adverse effects on renal (and muscular) glycolysis and gluconeogenesis, at least in rodents (Jones and Landon, 1967; Yoshida et al., 1970; Klahr et al., 1971; Fulgraff et al., 1972; Cohen and Little, 1976; Vinay et al., 1987; Dimitriadis et al., 1988; Dimitriadis et al., 1993; Amores et al., 1994). More recently, a potential functional link between NKCC2 and renal glucose fate has been suggested. Indeed, fructose-bisphosphate aldolase B, an enzyme involved in both gluconeogenesis and glycolysis, and primarily located in the kidneys, liver and intestines, may bind to, sequester, and reduce the functional expression of NKCC2 (Benziane et al., 2007) in a manner dependent of fructose 1,6-bisphosphate (F1,6BP), the enzyme’s substrate. Moreover, fructose, once activated to fructose-1-phosphate, can also serve as a substrate for aldolase B and has been shown to increase NKCC2 functional expression in the kidney (Ares et al., 2019). Although it is unknown whether NKCC2 can modulate the enzymatic activity of aldolase B in tubular cells, or if loop diuretics in general directly influence this interaction, these findings suggest a complex regulatory relationship with potential clinical implications. On one hand, there appears to be a negative regulatory link between renal gluconeogenesis and NKCC2 function. On the other hand, dietary fructose consumption, a potential contributor to MetS (Reungjui et al., 2007), is linked to NKCC2 function.

Although the role of NKCC2 in renal handling of glucose remains poorly defined, NKCC2A^KO^ mice developed several aspects of MetS including increased basal glycemia, glucose intolerance and insulin resistance (Kelly et al., 2019), but not hypertension, at least when mice were young (Oppermann et al., 2007). In addition, these mice showed enhanced glucose responses to alanine (Kelly et al., 2019), a substrate almost exclusively converted into glucose in the liver (Stumvoll et al., 1998; Meyer et al., 2002a; Mithieux et al., 2004; Mutel et al., 2011; Alsahli and Gerich, 2017; Sasaki et al., 2017). While these findings suggest increased hepatic *de novo* gluconeogenesis, the gluconeogenic response of NKCC2A^KO^ mice to exogenous pyruvate, which is converted into glucose in the liver, kidneys and small intestines (Stumvoll et al., 1998; Meyer et al., 2002a; Mithieux et al., 2004) remained normal (Kelly et al., 2019). Therefore, these observations suggest that NKCC2A^KO^ mice might have compromised renal gluconeogenesis. Moreover, aged NKCC2A^KO^ male mice developed overweight and consumed excessive food and water indicating that, unsurprisingly, the kidneys and other organs may contribute to the impaired glucose homeostasis observed in NKCC2A^KO^ mice (Kelly et al., 2019). In that regard, NKCC2 has been detected in other organs at much lower levels than those found in the kidneys, including small intestines (Xue et al., 2009) and hypothalamic regions of the brain (Konopacka et al., 2015). Even though the specific roles that NKCC2 in these organs may have in glucose homeostasis remain unexplored, the potential relevance of extrarenal NKCC2 is underlined by the following: *i*) the gluconeogenic capacity (Watford, 2005) of the small intestine supplies circulating glucose (Penhoat et al., 2014) and prevents obesity-related hepatic steatosis (Vily-Petit et al., 2020), *ii*) the hypothalamus plays a central role in endocrine integration of fuel homeostasis, control of water/energy intake and feeding behavior (Schwartz et al., 2000; Coll et al., 2007; Begg and Woods, 2013), and *iii*) as it has been known for a long time, diet and food intake affect renal gluconeogenesis and water balance (Author Anonymous, 1971a). Therefore, the metabolic phenotype of NKCC2A^KO^ mice likely stems from complex, age-dependent and long-term functional interactions between the brain, pancreatic islets, kidneys and intestines as well as other tissues where NKCC2 may be expressed, even in minimal quantities relative to the kidneys.

### 3.3 Effects of diuretics on renal glucose reabsorption

The kidneys utilize ∼10% of the total glucose used by the body in a daily basis, filtering 180 g of glucose per day, which is then almost entirely brought back into circulation (Ross et al., 1986; Alsahli and Gerich, 2017). Glucose is actively reabsorbed in the proximal convoluted tubule via the Na^+^-glucose transporter 2 (SGLT2), which couples the transport of the sugar with that of Na^+^ following its electrochemical gradient created by the Na^+^/K^+^ ATPase on the basolateral membrane of the tubular cells (see Figure 3B). Once inside the tubular cell, glucose is transported across the basolateral membrane into the peritubular capillaries by GLUT2 to reach back the bloodstream (Kanai et al., 1994). Importantly, SGLT2 is targeted by a class of highly efficacious drugs known as gliflozins, which reduce renal glucose reabsorption, thereby aiding in the management of glycemia and improving cardiovascular and metabolic health (Teo et al., 2021; Matthews, 2024). Notably, there has long been awareness that at least two loop diuretics, *i.e.*, furosemide and ethacrynic acid can moderately decrease glucose reabsorption in the proximal tubule (Bowman et al., 1973; Arruda et al., 1975; Boonjarern et al., 1977; Wen et al., 1978). However, the potential of loop diuretics (or thiazide and thiazide-like diuretics) to promote glycosuria through this or any mechanism remains uncertain. It is worth noting that SGLT2 inhibitors not only enhance glycemic control but also reduce hypertension and mitigate MetS in animal models co-administered with furosemide or hydrochlorothiazide (Rahman et al., 2016), as well as in clinical settings involving patients with chronic heart failure (Grodin and Tang, 2020; Ibrahim et al., 2020).

### 3.4 Effects of diuretics on liver and muscle glucose metabolism

Hepatic gluconeogenesis is a highly regulated process that serves as a backup for synthesizing glucose and glycogen from non-sugar sources (Zhang et al., 2018). Like the liver, muscle cells store glucose as glycogen. However, muscle glycogen is used locally for energy rather than being released into the circulation. During muscle activity, for instance, glycogen is broken down into glucose-6-phosphate for ATP production through glycolysis. This process can occur either aerobically or anaerobically, the latter leading to lactate production and release. Muscle-derived lactic acid is converted into alanine, transported to the liver, converted back to lactic acid and then used in *de novo* gluconeogenesis to synthesize glucose (see Figure 1). Glucagon effectively stimulates gluconeogenesis from amino acids and other non-carbohydrate substrates in the liver, but not in muscle, while insulin has the opposite effect, i.e., it inhibits hepatic glucose production and release (Puigserver et al., 2003; Adeva-Andany et al., 2019). Importantly, hepatic gluconeogenesis produces glucose-6-phosphate, which together with that produced from glycogen degradation (glycogenolysis) must be hydrolyzed by glucose-6-phosphatase in the endoplasmic reticulum to be released as glucose into the circulation (Cahill et al., 1959). Therefore, tissue glucose-6-phosphatase plays a major role in the maintenance of glycemia, particularly under fasting conditions.

Very little is understood about the metabolic effects that thiazides, thiazide-like and loop diuretics may have in hepatic glucose production and/or degradation. Nonetheless, early evidence did suggest that mechanisms sensitive to loop diuretics, possibly involving NKCC1 and/or KCCs, may contribute to the phosphorylation of numerous protein substrates in the liver (Lang et al., 1998). Among these proteins, is the serum- and glucocorticoid-dependent kinase (Waldegger et al., 1997), which is now recognized for its role in promoting hepatic insulin resistance (Zhou et al., 2021). Although this kinase was shown to regulate plasma membrane trafficking of NKCC2 *in vitro* (Fillon et al., 2001), the specific role of loop diuretics in developing hepatic insulin resistance remains unclear. It has been suggested that loop diuretics might contribute to insulin resistance in the liver (Schliess et al., 2001) and as such contribute to increased hepatic gluconeogenesis, while thiazides may exacerbate insulin resistance in general (Ramsay et al., 1992; Eriksson et al., 2008). Despite these findings, our current knowledge about the overall impact of diuretics on hepatic gluconeogenesis related to insulin resistance remains very limited.

Also poorly understood is the potential relationship that may exist between hepatocyte swelling in response to amino acids, the obligatory KCC-dependent K^+^/Cl^−^ extrusion, the resulting reduction in [Cl^−^]_i_ and glycogen synthesis via activation of the Cl^−^-dependent enzyme glycogen synthase phosphatase (Meijer et al., 1992). As Cl^−^ions can directly inhibit this enzyme (Meijer et al., 1992) as well as glucose-6-phosphatase (Pederson et al., 1998) one would expect that changes in [Cl^−^]_i_ may inversely correlate with glycogen biosynthesis or glucose production. However, like β-cells, the likely redundancy of diuretic-sensitive mechanisms involved in the regulation of hepatocyte [Cl^−^]_i_ makes it challenging to study the role of loop diuretics on hepatic glucose metabolism. In addition, the potential of hydrochlorothiazide to indirectly impair the function of pyruvate carboxylase by inhibiting carbonic anhydrase Vb (Kucharczyk et al., 2023) may have widespread physiological implications; this enzyme is widely distributed and plays an essential role in *de novo* gluconeogenesis and lipogenesis (Jitrapakdee and Wallace, 1999).

In comparison, virtually nothing is known about the metabolic effects that diuretics may directly have on muscle glucose homeostasis.

## 4 Conclusion

Limited often conflicting evidence mostly involving hydrochlorothiazide is still taken as proof of increased risk of T2D in hypertensive patients treated with any thiazide, thiazide-like or loop diuretics. Indeed, early studies have found that hydrochlorothiazide has the potential to influence various facets of glucose homeostasis, spanning from insulin secretion in the islets to the production of glucose in the liver and kidneys under diverse physiopathological conditions in humans and animal models. In addition, some studies examining the effects of bumetanide or furosemide on carbohydrate metabolism in humans have produced inconsistent results. Further, despite the persistent notion that all diuretics might have “diabetogenic properties”, long-term studies in preclinical animal models are still missing and many questions remain unanswered regarding the mechanisms whereby these drugs may exert their metabolic effects under different chronic contexts. Untangling the potential effects of these diuretics on fuel homeostasis is additionally complicated by the intricate relationships among all components of the MetS, glucose intolerance (often confused with prediabetes), T2D, hypertension, the specific diuretic treatment and the functional redundancy that may exist among diuretic-sensitive targets. Whilst certain studies do hint at possible direct effects of thiazide, thiazide-like and loop diuretics on glucose homeostasis, and that its control is apparently beneficial for some aspects of the MetS, it is clear that additional research is necessary to fully understand the specific mechanisms involved and the potential clinical implications that they may have in hypertensive individuals with chronic metabolic conditions.
